# Clinical prospects of WRN inhibition as a treatment for MSI tumours

**DOI:** 10.1038/s41698-022-00319-y

**Published:** 2022-11-15

**Authors:** David A. Morales-Juarez, Stephen P. Jackson

**Affiliations:** 1 Wellcome and Cancer Research UK Gurdon Institute, and Department of Biochemistry, University of Cambridge, Cambridge, UK; 2 Present Address: Cancer Research UK Cambridge Institute, University of Cambridge, Cambridge, UK

**Keywords:** Targeted therapies, Drug development, Cancer therapy, Biomarkers, Cancer therapeutic resistance

## Abstract

The discovery of synthetic lethal interactions with genetic deficiencies in cancers has highlighted several candidate targets for drug development, with variable clinical success. Recent work has unveiled a promising synthetic lethal interaction between inactivation/inhibition of the WRN DNA helicase and tumours with microsatellite instability, a phenotype that arises from DNA mismatch repair deficiency. While these and further studies have highlighted the therapeutic potential of WRN inhibitors, compounds with properties suitable for clinical exploitation remain to be described. Furthermore, the complexities of MSI development and its relationship to cancer evolution pose challenges for clinical prospects. Here, we discuss possible paths of MSI tumour development, the viability of WRN inhibition as a strategy in different scenarios, and the necessary conditions to create a roadmap towards successful implementation of WRN inhibitors in the clinic.

## Synthetic lethality – accelerating precision oncology

Precision oncology aims to tailor cancer treatments to the specific biology of patients and their underlying tumours through genomic profiling, biomarker-mediated stratification, and choice of selected therapies^1^. Therefore, the potential for precision oncology hinges on improvement of profiling strategies, identification of therapeutically relevant biomarkers, and development of novel drugs to selectively target different cancer types^2^. Advances in next-generation sequencing technologies, such as deep-sequencing approaches and epigenomic profiling, are constantly improving our ability to genetically map tumour heterogeneity and enable better patient stratification^3–5^. However, the discovery of actionable candidates for targeted therapy and downstream drug development processes is time consuming, expensive, and often unsuccessful^6,7^. Consequently, identification of molecular signatures that can be precisely targeted by potent and specific drugs with a high likelihood of clinical success is paramount to make drug development a worthwhile investment^8,9^.

The ultimate goal of anti-cancer drug development is the discovery of chemicals that can eliminate cancer cells without harming the patient’s normal cells^10^. To this end, pharmaceutical companies and research laboratories alike are investing major resources in identifying actionable synthetic lethal interactions^11^. Synthetic lethality occurs when simultaneous mutations in two genes causes cell death, but a single mutation in either gene is viable^12,13^. Due to the nature of these interactions, inhibiting the products of genes that have a synthetic lethal relationship with prevalent genetic mutations in cancer cells should specifically kill cancer cells, while sparing normal cells^14^. Exploiting the selective vulnerabilities of cancer cells bearing specific mutations and/or pathway dysfunctions, through inhibition of their synthetic lethal partners has produced various levels of success in the clinic^15–17^. The most established and successful of these endeavours, thus far, is the development of Poly (ADP-ribose) polymerase (PARP) inhibitors to selectively target BRCA1/2 mutated cancers and other cancers with underlying defects in DNA repair by homologous recombination^18–21^. Such developments have fuelled focused studies and large-scale projects to systematically map cancer-specific dependencies^22^.

## Mismatch repair deficiency leads to microsatellite instability

DNA mismatch repair (MMR) is a conserved mechanism that contributes to maintenance of genome stability by removing errors generated during DNA replication, long-tract DNA repair synthesis, and recombination^23^. Germline or somatic mutations and epigenetic alterations in the genes of MMR components lead to a hypermutator phenotype characterized by cancer predisposition and high genomic instability, particularly at repetitive regions of the genome known as microsatellites^24^. Microsatellites, or short-tandem repeats, are short (1–6 base pair) repetitive DNA sequences distributed along coding and non-coding regions that constitute approximately 3% of the human genome^25^. Due to their repetitive nature, microsatellites are prone to DNA polymerase slippage events, producing INDELs (insertions and deletions) that are mainly recognized and repaired by MMR^26^. Consequently, MMR deficiencies lead to a phenotype termed microsatellite instability (MSI), characterized by the accumulation of repeat-length alterations at microsatellite regions^27^. Detection of MSI is clinically relevant, since patients with MSI cancers have a better overall prognosis and reduced metastatic potential compared to patients with microsatellite stable (MSS) cancers^28^. This seems, in part, to reflect the high mutational burden of MSI tumours causing production of neoantigens that increase immunogenicity and sensitivity to immune checkpoint inhibitors^29–32^. Nevertheless, a significant proportion of MSI tumours do not respond, or evolve resistance, to immunotherapy and chemotherapy, highlighting the need for more and improved targeted and combinatorial treatments^33–35^. Key aspects in the relationship between MMR deficiency and cancer are outlined in Box 1 and current MSI detection methods are shown in Table 1.

**Table 1:** MSI detection methods.

Method	Accuracy	Property tested
Fluorescent multiplexed PCR and capillary electrophoresis^66^	~100% (standard)	Instability in 5 distinct microsatellites
Next-generation sequencing^67^	~92–94%	Instability in ~100 distinct microsatellites
Single-molecule molecular inversion probes^68^	~90–95%	Instability in 111 distinct microsatellites
Immunohistochemistry^69^	~95% (standard)	Presence of MMR proteins

Box 1 DNA mismatch repair deficiency and cancer **Lynch syndrome**

Heterozygous germline alterations in certain DNA mismatch repair genes (*MLH1*, *MSH2*, *MSH6*, and *PMS2*) give rise to Lynch syndrome, also known as hereditary nonpolyposis colorectal cancer (HNPCC)^70^. Lynch syndrome is an autosomal dominant disorder that accounts for 3–5% of colorectal cancer cases and approximately 2.5% of endometrial cancer cases^71,72^. Diagnosing Lynch syndrome patients is clinically relevant since these patients have an 80% lifetime risk of developing colorectal cancer, and an increased risk of developing endometrial, ovarian, urinary tract, and gastric cancers among others^73^. It is estimated that Lynch syndrome affects 1/300 to 1/500 individuals in the general population, making it one of the most common genetic predispositions to cancer^74^.

**Constitutional mismatch repair deficiency syndrome**

Constitutional mismatch repair deficiency (CMMRD) arises from homozygous alterations in DNA mismatch repair genes and is characterized by a drastic predisposition to cancer^75^. In contrast to the relatively low prevalence of tumours in Lynch syndrome patients in the early stages of life, almost all individuals with CMMRD develop cancer in childhood and early adolescence, with a generally poor prognosis^76,77^. The most common cancers developed by CMMRD patients are colorectal, brain, and blood cancers, and CMMRD patients have a high likelihood of developing multiple cancers throughout their lives^78^.

**Microsatellite instability in cancer**

Microsatellite instability (MSI) is present in at least 27 different tumour types, with the prevalence of MSI ranging from ~31% in endometrial carcinoma to 0.25% in glioblastoma multiforme^79^. Moreover, MSI is detected in ~15% of colorectal cancers; approximately 3% of these are associated with Lynch syndrome and the other 12% are caused by somatic alterations in MMR, most often promoter hypermethylation of the *MLH1* gene^80,81^. Importantly, MSI is present in most tumours associated with Lynch syndrome^55,56^. Cancers with MSI show aggressive histological features, but paradoxically favourable prognosis^82^.

## WRN inactivation is synthetic lethal with MSI

In 2019, a set of high-impact publications independently unveiled a therapeutically promising synthetic lethal relationship between the RecQ-like family helicase protein, WRN, and MSI tumours^36–39^. WRN is a multifunctional enzyme with helicase and exonuclease activities and plays roles in various cellular processes crucial for the maintenance of genome stability, including DNA replication, transcription, DNA repair, and telomere maintenance^40–43^. Through CRISPR-Cas9 and RNAi screens, WRN was identified as the top hit for preferential dependency in MSI but not MSS cancer cell lines^36,37,39^. Further analysis revealed that WRN depletion causes cell cycle arrest, DNA damage, mitotic defects, chromosome shattering, and apoptosis specifically in MSI cells^36–39^. Moreover, WRN depletion reduced xenograft growth and tumour formation in mice transplanted with MSI cells^39^. Strikingly, acute depletion of various MMR components did not induce WRN dependency in MSS cells; conversely, genetic rescue experiments in MSI cells re-introducing the missing MMR component failed to rescue the synthetic lethal relationship^36^. These experiments suggested that the WRN synthetic lethal relationship develops via ensuing mutational consequences of MMR dysfunction rather than through MMR deficiency per se. Additionally, dissection of the various enzymatic activities of WRN using loss-of-function mutations within the helicase domain, exonuclease domain, or both, demonstrated that WRN dependency in MSI cells is linked only to its helicase function^37^.

## Mechanistic insights into the WRN dependency of MSI tumours

Microsatellites can adopt non-B form DNA secondary structures in a sequence- and length-dependent manner^44^. Interestingly, one of the initial publications unveiling the synthetic lethality between WRN and MSI hypothesized that the potential mechanism driving this dependency was an increase in noncanonical secondary DNA structures that require WRN for their resolution^38^. Indeed, a seminal publication by van Wietmarschen et al. in *Nature* later demonstrated that TA-dinucleotide repeats are highly unstable in MMR deficient cells and undergo large-scale expansions in this setting, ultimately forming non-B form DNA secondary structures^45^. In *Escherichia coli* and yeast, expanded (TA)_n_ repeats can form cruciform structures when their length exceeds roughly 20 repeat units^46,47^. Furthermore, long (TA)_n_ tracts cause replication fork stalling and chromosome fragility at common fragile sites (CFSs)^48,49^. Importantly, WRN can resolve various non-B DNA substrates, including forks, flaps, bubbles, Holliday junctions, displacement loops (D-loops), and G-quadruplexes^50,51^. Accordingly, WRN depletion was found to induce replication fork collapse and DNA double-strand break formation precisely at expanded (TA)_n_ repeats in MSI cells^45^. Mechanistically, expanded (TA)_n_ microsatellites likely form cruciform structures that cause replication fork stalling, activating the apical ATR kinase, and causing the recruitment of WRN to resolve these structures via its helicase activity^45^. However, in the absence of WRN, expanded (TA)_n_ repeats are unresolved and cleaved by the structure-specific endonucleases MUS81-EME1 and SLX4, leading to extensive chromosome shattering and ensuing cell death^45^. These findings suggest that WRN is uniquely able to resolve non-B form DNA secondary cruciform structures that form from (TA)_n_ expansions in MSI cells and provide a mechanistic explanation for the synthetic lethal relationship between WRN and MSI.

## Clinical potential of WRN inhibitors – lost in translation?

The synthetic lethal relationship between WRN and MSI has nurtured interest by academic groups and companies to develop WRN inhibitors to selectively target MSI cancers, with some drug development programmes already underway^11,52^. Importantly, WRN dependence appears to be conserved within heterogenous MSI tumour models, albeit only when MSI is derived from MLH1 or MSH2 deficiencies^53^. Of note, the small number of MSI models that can tolerate WRN loss appear to lack the MSI genomic (TA)_n_ repeat expansion characteristics that invoke WRN dependence, supporting the likely clinical relevance of this relationship^53^. These findings suggest WRN dependency is influenced by the underlying MMR gene altered and the degree of MMR deficiency conferred. Collectively, mounting evidence supports the potential of future WRN inhibitors to selectively treat a subset of MSI tumours. Nevertheless, the data gathered so far might not be painting the full picture.

All reported work studying WRN dependence in MSI tumours, has been done in cellular models that fail to capture the full extent of the intratumour heterogeneity observed in patients. Understanding clonal diversity within MMR deficient cancers throughout their evolution is crucial to be able to accurately stratify patients, develop robust chemotherapeutic strategies, and attempt to avoid resistance to therapy^54^. A key issue to note is that, while MMR deficiency nurtures MSI, (TA)_n_ repeat expansions, and tumourigenic mutations in tumour suppressors and oncogenes, these outcomes are largely stochastic and independent of one another. Indeed, MMR deficiency does not invariably, at the cellular level, lead to MSI or cancer development, as evidenced by experimental studies showing that acute MMR dysfunction does not cause MSI or WRN dependence, the subset of Lynch syndrome patients that do not develop cancer in their lifetimes, and the presence of MMR deficient cancers that are not MSI^55–58^. Consequently, the spatiotemporal development of MSI with (TA)_n_ repeat expansions and cancer in MMR deficient clones in a tissue context is still poorly understood and is an elusive, but fundamental, issue to explore.

**Fig. 1:**
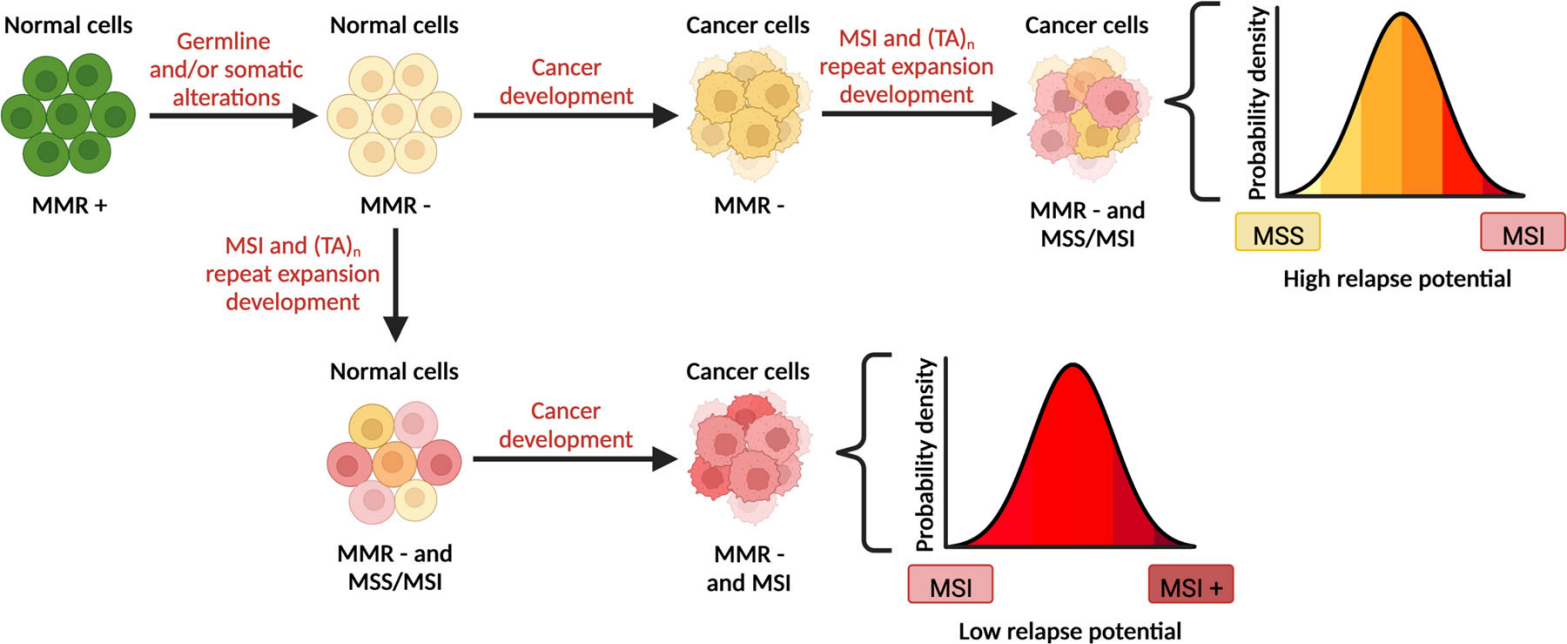
Temporal differences in MSI and cancer development might lead to contrasting clinical outcomes upon WRN inhibition. Schematic outlining the possible paths towards developing MSI tumours with (TA)_n_ repeat expansions that can be targeted with WRN inhibitors. Acquiring MMR deficiency, through germline and/or somatic alterations in MMR genes, is a necessary first step towards MSI and tumour development. MMR deficient cells can become cancerous before developing MSI and (TA)_n_ repeat expansions (top branch) or develop MSI and (TA)_n_ repeat expansions before becoming cancerous (bottom branch) potentially leading to different clinical outcomes. The therapeutic window of WRN inhibitors ultimately depends on MSI status and the degree of (TA)_n_ repeat expansions. Microsatellite stable (MSS) and MSI cells with (TA)_n_ repeat expansions are highlighted in yellow and red respectively. Figure was created with BioRender.com.

The likely impact of future WRN inhibitors will ultimately be in MSI cells that carry the molecular signature of (TA)_n_ repeat expansions. Consequently, the prevalence of this molecular signature within MSI tumours and normal tissues is important to investigate and define. In this regard, it is critical to discuss the potential scenarios of MSI and cancer development in cells with somatically acquired or germline MMR deficiency (Fig. 1). Cells that have acquired MMR deficiency can gather tumourigenic mutations and become cancerous before developing MSI (Fig. 1, top branch), or develop MSI before becoming cancerous (Fig. 1, bottom branch). On one hand, if cancerous cells arise first followed by MSI development, the overall population of cells in the ensuing tumour would have various degrees of microsatellite instability ranging from MSS to MSI (Fig. 1, top branch). Since WRN inhibition would be effective only on the MSI cells that have developed (TA)_n_ repeat expansions, a considerable proportion of the cells in such a tumour could be unaffected, likely causing failure to respond or relapse. Nevertheless, combinatorial therapies with immune checkpoint inhibitors or other chemotherapies might still enable favourable outcomes. On the other hand, if MSI development occurs first followed by cancer evolution from an MSI clone, the entire cancer cell population would be MSI and, thereby, WRN dependent (Fig. 1, bottom branch). Although WRN inhibition in this context might effectively eliminate the cancer cells, full cancer eradication would only ensue if the MSI cells had accumulated sufficient (TA)_n_ repeat expansions to render them WRN dependent before becoming transformed. The extent to which such a scenario exists clinically, however, remains to be established. Crucially, even a small number of MSI cancer cells with low levels of (TA)_n_ repeat expansions could lead to tumour relapse. In this regard, it would be interesting to investigate whether MSI cancer cells with (TA)_n_ repeat expansions have the potential to evolve resistance to WRN inhibition, by altering the length and/or sequences of the (TA)_n_ repeat expansions, by inactivating MUS81-EME1 and/or SLX4 which may alleviate the DSB formation and chromosomal shattering, or by up-regulating genome stability processes, perhaps involving promiscuous DNA helicases that might compensate for WRN inhibition in this context. Importantly, reversion mutations of the underlying MMR defect would not confer resistance to WRN inhibition since the MSI phenotype would already be established^59^.

## Paving the way – towards a roadmap for clinical success

**Fig. 2:**
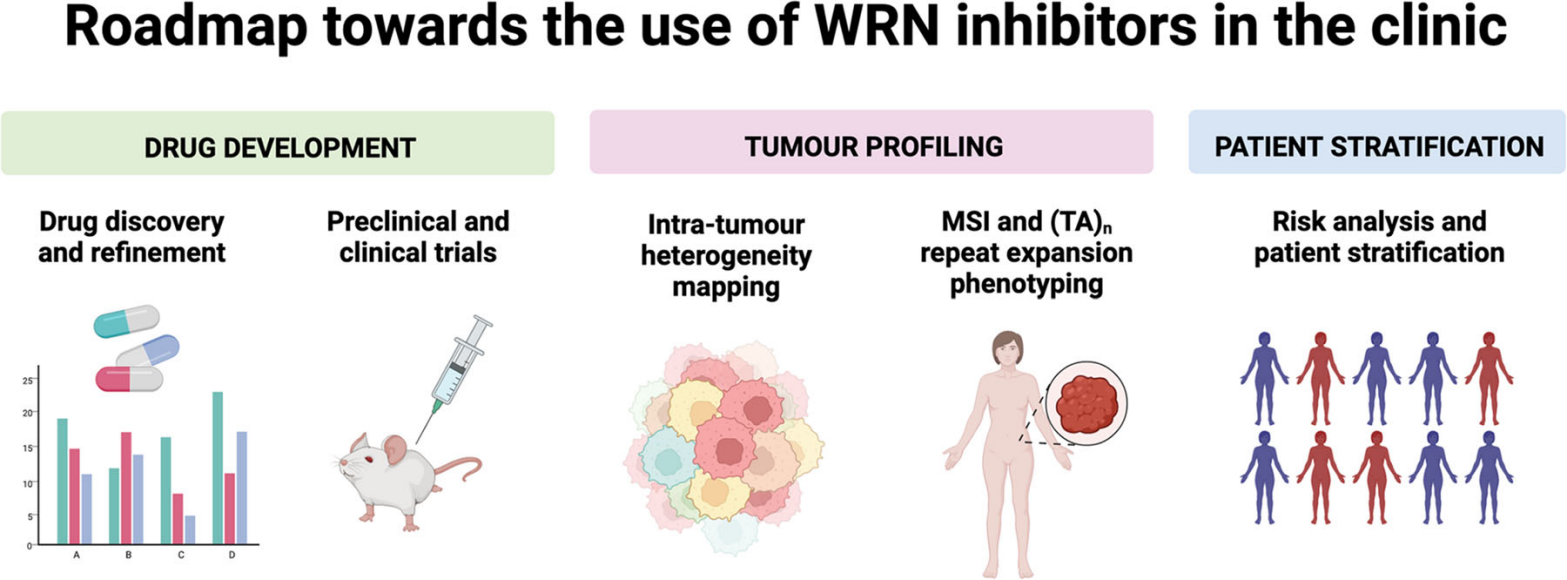
Roadmap towards the use of WRN inhibitors in the clinic. Future clinical success of WRN inhibitors hinges on three pillars: drug development, tumour profiling, and patient stratification. Development and refinement of WRN inhibitors that are potent, selective, bioavailable, and relatively safe is an absolute requirement. Improvements in genetic and epigenetic tumour profiling through deep sequencing, liquid biopsies, and other technologies would provide a clearer picture of intra-tumour heterogeneity. Moreover, MSI testing will need to be coupled with (TA)_n_ repeat expansion profiling to predict the potential of WRN inhibition. Finally, risk analysis through cancer modelling and biomarker identification will invariably improve patient stratification. Figure was created with BioRender.com.

The scenarios of MSI tumour development outlined above pose real challenges in the clinic that could seriously limit the prospects for effective use of WRN inhibitors in treating a subset of MSI cancers. Furthermore, the initial hurdle of developing a suitable compound for clinical exploitation which is specific, potent, and bioavailable must not be understated^60^. Moreover, the lack of clarity and specificity when diagnosing a complex phenotype like MSI with (TA)_n_ repeat expansions, gives rise to various issues that need to be addressed before fully grasping WRN dependence in this context. Mapping of intratumour heterogeneity and normal tissue heterogeneity regarding MSI and (TA)_n_ repeat status, will invariably provide clarity and enable better patient stratification for the future use of WRN inhibitors. Recent advances in tumour mapping and identification using liquid biopsies, deep sequencing, and bioinformatic modelling of cancer evolution will be useful tools towards predicting patient outcomes^61–65^. In sum, the roadmap towards clinical applications of WRN inhibitors is paved by roadblocks in drug development, tumour profiling, and patient stratification. Surmounting these will be a highly sought and worthy goal for the researchers, drug developers, and clinicians, whose collective efforts will be necessary to successfully deliver WRN inhibitors to the patients who would derive the most benefit (Fig. 2).

## Data Availability

Not applicable to this article as no datasets were generated or analyzed.
